# The Cost of Provisions in Institutions.—V

**Published:** 1904-05-14

**Authors:** 


### THE COST OF PROVISIONS IN INSTITUTIONS.—V.

By the Author of "Hospital Expenditure—The Commissariat."

(*Continued from page* 89.)

### THE GREAT NORTHERN CENTRAL HOSPITAL
(159 Available Beds).

The following figures give the cost of provisions per bed in this hospital for the last five years:—

| Year | Cost per occupied bed. | Average number of occupied beds | Number of available beds |
|---|---|---|---|
| | £ | | |
| 1902 ... ... | 20 | 137 | 159 |
| 1901 ... ... | 19¼ | 134 | 159 |
| 1900 ... ... | 18¾ | 143 | 159 |
| 1899 ... ... | 18 | 130 | 146 |
| 1898 ... ... | 18¾ | 108 | 134 |

These figures represent the total cost for provisions of all the inmates of the hospital, doctors, nurses, male and female servants, divided by the average number of occupied beds.

For the year 1902 the numbers boarded, other than patients, were as follows, those on partial board reckoning as half. The percentage of non-patients boarded is 38.

| | Number | Proportion of occupied beds to each | Number of available beds to each |
|---|---|---|---|
| Medical and other officers | 9 | 15 | 17·6 |
| Nursing staff... | 41 | 3 | 3·8 |
| Male servants | 14 | 11 | — |
| Female servants | 19 | 7 | 8 |
| | — | — | — |
| Total ... | 83 | 1·6 | 1·9 |

The numbers vary slightly from time to time, and the average number of those other than patients boarded in the hospital during the last three years is 85. There is no house laundry, hence no laundry-maids, and the consumption of ice and mineral waters is not sufficient to make it worth while to make these articles on the premises, so that in these departments no service is required. It is these points which tend to lower the average cost of provisioning the smaller hospitals, since the economies effected by laundry or ice machine would benefit other departments while increasing the daily average boarded.

| Year | Number of occupied beds | Number of available beds | Total cost of provisions | Cost of patients at £11 10s. a year | Average cost of provisions exclusive of patients | |
|---|---|---|---|---|---|---|
| | | | | | Per available bed | Per occupied bed |
| | | | £ | £ | £ | £ |
| 1902 | 137 | 159 | 2,758 | 1 575 | 7¼ | 8½ |
| 1901 | 134 | 159 | 2 597 | 1.541 | 6½ | 8 |
| 1900 | 143 | 159 | 2.674 | 1,644 | 6½ | 7 |
| 1899 | 130 | 146 | 2 358 | 1,495 | 5¾ | 6½ |
| 1898 | 108 | 134 | 2,050 | 1,242 | 6 | 7½ |

The above table gives the average cost of provisions per available bed when the patient's board, which averages between 4s. 3d. and 4s. 6d. a head weekly, has been deducted.

The system of accounts in this hospital, of which we hope later on to give a brief synopsis, shows three strands of expenditure for patients, paying patients, and household. The cost of patients and paying patients is not materially different, averaging 4s. 3d. to 4s. 6d. per head per week.

The cost of the household, reckoning together the officers' table, the nurses' table, and that of the male and female servants, works out at from 5s. 3d. to 5s. 6d. per head per week.

Analysing the published report of expenditure in 1902 it appears that the following was the average cost per bed and per head for the various items of provisions.

| | |
|---|---|
| Daily average of patients ... ... ... | 137 |
| Daily average of non-patients ... ... ... | 83 |
| Total daily average boarded in hospital ... | 220 |

| — | Total cost | Cost per occupied bed | Cost per head (all inmates) |
|---|---|---|---|
| | £ | £ s. | £ s. |
| Meat ... ... ... | 996 | 7 5 | 4 10 |
| Fish, Poultry, etc. ... | 181 | 1 6 | 16 |
| Butter, Cheese, etc. ... | 271 | 2 0 | 1 4 |
| Eggs ... ... ... | 122 | 18 | 11 |
| Milk ... ... ... | 572 | 4 3 | 2 12 |
| Bread, Flour, etc. ... | 206 | 1 10 | 18 |
| Grocery ... ... ... | 294 | 2 2 | 1 6 |
| Vegetables ... ... | 90 | 13 | 8 |
| Malt Liquors ... ... | 23 | 3 | 2 |
| Ice and Mineral Waters | 60 | 8 | |
| Wine and Spirits ... | 153 | 1 2 } patients only | |

We desire to concentrate attention rather on the rate of consumption than on the price of these articles.

In an average week it was found that 553 lb. of meat, as received from the butcher, including bone, were required for 140 patients, the average being just under 4 lb. a week each. This includes the beef used in making beef-tea, and in the same average week the amount of beef-tea required was 528 pints or 3·7 pints a head.

It is worth noting that this quantity of beef-tea made in the ordinary way with a pound of meat to a pint of water would have absorbed nearly the whole weight of meat found sufficient for all the patients during the week. The plan which is adopted in the hospital is to keep a large stock-pot going, in which all the odds and ends of the meat, trimmed off before cooking, are utilised. Here bones, gristle and fat contribute their share of nourishment. Mutton is used as well as beef, and the broth, duly strained and skimmed, is of excellent quality and of far more appetising flavour than if made entirely with the ordinary shin of beef.

The plan which is adopted here of buying sheep in the carcase and cutting them up on the premises, lends itself particularly well to the stock-pot system, under which the hospital reduces the heavy expense of beef-tea to a minimum.

Turning now to the household it is found that the average consumption of meat for the 83 persons composing it is 470 lb., or an average of 5·6 lb. per head; this works out at exactly 14 oz. a day per head, which is the average amount usually reckoned in allowance-sheets for adult persons engaged in active work.

With regard to the patients it is more difficult to assign an exact average per day, the number on milk or low diet, excluding either meat or beef-tea, being found to vary widely according to the class of case admitted. If all these patients were receiving meat or beef-tea in this particular week the average quantity of meat per head amounts to 9 oz. per day.

The quantity of milk required in a given week for 140 patients was 1,588 pints or 1·6 pints per head per day. In practice it is found that an average of 1½ pints a day is consumed by surgical patients and of 2 pints by medical patients. This amount is calculated daily and the right allowance is distributed to each ward. There is, perhaps, no direction in which waste is more commonly permitted in hospitals than in the distribution of milk, and this can hardly be checked in any other way than by limiting the ward sister to what has been found by experience to be a safe average. It is understood that convalescent patients receive less, while those on low diet may require double the above averages.

The household consumption of milk amounted in an average week to 798 pints, or 4½ quarts per head. This allows for a large proportion of the staff using milk as a beverage, and the extent to which milk is replacing beer as a stock beverage in institutions is matter for congratulation, when the comparative food-value of these drinks is considered.

The small expenditure on malt liquor which, as we have seen, amounts to about 2s. per head in the course of a year, is an increasingly common feature in hospital accounts. Convalescent patients who are accustomed to drink beer are not debarred from it, but beer-drinking is by no means the rule. Among members of the staff the use of malt liquor is small indeed, milk or mineral water being almost universally preferred.

The relatively small expenditure on fish and poultry, amounting to about 16s. a head a year, is a special feature in the accounts. Great economy is effected in this direction by purchasing direct from Grimsby, whence a varied supply is obtained of high quality at about half the ordinary cost of fish supplied in the usual way in ready-trimmed "diets."

The kitchens at the Great Northern Central Hospital are on the top floor and are particularly light and airy with an uninterrupted view over half London. They are fitted with the best modern apparatus and are connected by a lift with the basement, where all stores are received and where the meat is cut up.

